# 1-Palmitoyl-2-Linoleoyl-3-Acetyl-rac-Glycerol (PLAG) Mitigates Monosodium Urate (MSU)-Induced Acute Gouty Inflammation in BALB/c Mice

**DOI:** 10.3389/fimmu.2020.00710

**Published:** 2020-04-24

**Authors:** Su-Hyun Shin, Jinseon Jeong, Joo Heon Kim, Ki-Young Sohn, Sun Young Yoon, Jae Wha Kim

**Affiliations:** ^1^Division of Systems Biology and Bioengineering, Cell Factory Research Center, Korea Research Institute of Bioscience and Biotechnology, Daejeon, South Korea; ^2^Department of Functional Genomics, University of Science and Technology, Daejeon, South Korea; ^3^Division of Global New Drug Development, ENZYCHEM Lifesciences, Seoul, South Korea; ^4^Department of Pathology, EulJi University School of Medicine, Daejeon, South Korea

**Keywords:** neutrophil, chemokine, GPCR, gouty inflammation, receptor trafficking

## Abstract

Acute gouty arthritis is an auto-inflammatory disease caused by the deposition of monosodium urate (MSU) crystals in joints or tissues. Excessive neutrophil recruitment into gouty lesions is a general clinical sign and induces a pain phenotype. Attenuation of successive periods of neutrophil infiltration might be a beneficial approach to achieve therapeutic efficacy. In this study, the activity of 1-palmitoyl-2-linoleoyl-3-acetyl-rac-glycerol (PLAG) in attenuation of excess neutrophil infiltration was assessed in gout-induced lesions of BALB/c mice. Neutrophil infiltration in MSU-induced gouty lesions was analyzed using immunohistochemical staining. ELISA and RT-PCR were used to measure attenuation of expression of the major neutrophil chemoattractant, CXC motif chemokine ligand 8 (CXCL8), in a PLAG-treated animal model and in cells *in vitro*. The animal model revealed massive increased neutrophil infiltration in the MSU-induced gouty lesions, but the PLAG-treated mice had significantly reduced neutrophil numbers in these lesions. The results also indicated that the MSU crystals stimulated a damage-associated molecular pattern that was recognized by the P2Y6 purinergic receptor. This MSU-stimulated P2Y6 receptor was destined to intracellular trafficking. During intracellular endosomal trafficking of the receptor, endosome-dependent signaling provided expression of CXCL8 chemokines for neutrophil recruitment. PLAG accelerated initiation of the intracellular trafficking of the P2Y6 receptor and returning the receptor to the membrane. This process shortened the intracellular retention time of the receptor anchoring endosome and subsequently attenuated endosome-dependent signaling for CXCL8 expression. These study results suggested that PLAG could be used for resolution of acute inflammation induced in gout lesions.

## Introduction

Gouty arthritis is an inflammatory joint disease that most often occurs in men over 40 years of age (1). Worldwide, the incidence of this condition is 0.08–0.13% in men and 0.03% in women (2). Gout is accompanied by tenderness, erythema, redness, swelling, pain, and fever in periarticular tissues or in joints with accumulated monosodium urate (MSU) crystals (3, 4). MSU crystal-induced inflammatory disorder is well-represented in the gout disease model; neutrophil infiltration into the inflamed joints is the most distinctive phenotype (5). MSU crystal-induced intracellular signaling generates acute joint inflammation and mediates neutrophil migration and activation (6). The progression of sterile inflammation in acute gouty arthritis is promoted by an auto-amplification loop of the inflammation (7).

Activation of the NACHT, LRR, and PYD domains-containing protein3 inflammasome leading to interleukin-1β (IL-1β) release is a putative pathogenesis of acute gouty inflammation triggered by MSU crystals (8, 9). High concentration of CXC motif chemokine ligand 8 (CXCL8/IL-8) occurs in the synovial fluid of acute gout patients (10). CXCL8 is a well-known mediator of inflammation; it has key roles in neutrophil recruitment, neutrophil degranulation, and neutrophil extracellular traps (11, 12). A rabbit model of MSU-induced arthritis revealed that IL-8 neutralizing antibody inhibits neutrophil influx (13). Mice with knockout of the CXCR2 chemokine receptor, CXCL8 receptor, have reduced levels of MSU crystal-induced neutrophilic inflammation (13).

MSU induces gouty inflammation through recognition of its cognate receptors as an extracellular damage-associated molecular pattern (DAMP) (14, 15). MSU is directly recognized by the purinergic receptor, P2Y6, which is a G-protein-coupled receptor (GPCR). P2Y6 induces the production of CXCL8 and IL-1β via P2Y6 receptor signaling (16, 17). GPCRs exhibit classical signaling that is restricted to cell membranes and persistent signaling that depends on internalization of the GPCR bound to β-arrestins (18). Receptor trafficking is important for control of GPCR signaling and is regulated by multiple cellular proteins [e.g., α-arrestin, β-arrestin, clathrin, and G protein receptor kinases (GRKs)] (18–22).

Intracellular trafficking of GPCRs also activates intracellular signaling, but the MSU/P2Y6 receptor/CXCL8 signal axis is not fully understood. TIR-domain-containing adapter-inducing interferon-β (TRIF)-dependent signaling is generally mediated by the endocytosis of Toll-like receptor 4 (23, 24). One study found that an interaction between TRIF and protease-activated receptor 2 (PAR2, a GPCR) occurs during interferon regulatory factor 3 (IRF3) activation (25). CXCL8 is expressed by a TRIF/IRF3-dependent pathway (26, 27).

The compound 1-palmitoyl-2-linoleoyl-3-acetyl-rac-glycerol (PLAG) is a mono-acetyl-diacylglycerol isolated from the antlers of sika deer. It is chemically synthesized from glycerol, palmitic acid, and linoleic acid (28). PLAG reduces lipopolysaccharide-induced macrophage inflammatory protein 2 (MIP-2) and CXCL8 secretion (29). PLAG improves hepatic injury in concanavalin A-treated mice (9), and gemcitabine-induced neutropenia (30, 31) and oral mucositis in hamster and mouse models (32), via modulation of neutrophil migration. In this study, the mitigating effects of PLAG in MSU-induced acute gouty inflammation through modulation of neutrophil infiltration were investigated. The roles of PLAG in the regulation of CXCL8 expression and P2Y6 intracellular trafficking were also examined in MSU-treated THP-1 cells.

## Materials and Methods

### Animal Model Experimental Design

The animal experiments were performed using 9- to 11-week-old male BALB/c mice (Koatech Co., Pyongtaek, Republic of Korea). The mice were housed under standard conditions maintaining a 12-h dark/light cycle and an *ad libitum* supply of food and water. The mice were divided into three groups. Each group consisted of five animals: Group 1: control mice, administered 50 μl PBS (vehicle control) via injection into the left footpad of each mouse, followed by oral administration of 100 μl PBS alone every day for 3 days; Group 2: a suspension of 1 mg MSU crystal in 50 μl of PBS was injected into the left footpad of each mouse, followed by oral administration of 100 μl PBS every day for 3 days; Group 3: a suspension of 1 mg MSU crystal in 50 μl of PBS was injected into the left footpad of each mouse, followed by oral administration of 250 mg/kg/day of PLAG (Enzychem Lifesciences Co., Daejeon, Republic of Korea) every day for 3 days. MSU crystal-injected footpad swelling was calculated based on before and after footpad thickness measured using a Digimatic Caliper (Mitutoyo Corporation, Kawasaki, Japan). Mice were anesthetized with 2,2,2-Tribromoethanol (150 mg/kg, sigma-Aldrich, St. Louis, MO, USA) by intraperitoneal injection after foot pad swelling evaluation and were sacrificed after photography. Dissected footpads were fixed directly in 10% buffered formalin for H & E staining and IHC and stored directly in Tri-RNA Reagent for RT-PCR.

### Preparation of Monosodium Urate Crystals

The MSU crystals were prepared using crystallization of a supersaturated solution of uric acid (Sigma, USA). First, 250 mg of uric acid was added to 45 ml distilled water containing 300 μl of 5 M NaOH. The solution was boiled until the uric acid was completely dissolved and then passed through a 0.45 μM filter. Next, 1 ml of 5 M NaCl was added to the hot solution; the solution was then stored at 26°C to allow crystallization. After 10 days, the MSU crystals were washed with ethanol and allowed to air dry under sterile condition (33).

### Hematoxylin and Eosin Staining and Immunohistochemistry

Footpad samples obtained 3 days after MSU crystal injection were immediately fixed in 10% buffered formalin for 24 h at room temperature. Formalin-fixed paraffin sections (4-μm thickness) were stained using hematoxylin and eosin. Immunohistochemistry was performed to detect neutrophils at MSU crystal-injected tissue. Serial 4-μm thick sections of the footpads were mounted on charged glass slides (Superfrost Plus; Thermo Fisher Scientific, Rochester, NY, USA). The tissue sections were deparaffinized and treated using 3% hydrogen peroxide in methanol to eliminate endogenous peroxidase activity. They were then incubated with 1% bovine serum albumin (BSA) to block non-specific binding at room temperature for 30 min. The sections were incubated with primary rat anti-neutrophil antibody (1:200; NIMP-R14, Thermo Fisher Scientific Inc.) at room temperature for 2 h. After washing with Tris-buffered saline, the slides were incubated with the secondary antibody, horseradish peroxidase-conjugated goat-anti-rat IgG (1:250; Santa Cruz Biotechnology, Dallas, Texas, USA), for 30 min at room temperature. The resulting images were examined using light microscopy (Olympus) and neutrophil staining area was calculated using image J program.

### Cell Culture

We established an *in vitro* cell culture system using the human monocytic cell line THP-1 (ATCC, TIB-202), which was grown in RPMI 1640 (Welgene, Gyeongsan-si, South Korea) supplemented with 2-mercaptoethanol to a final concentration of 0.05 mM, 10% fetal bovine serum (FBS; Tissue Culture Biologicals, CA, USA) and penicillin/streptomycin as recommended by ATCC. The growth of the cells was maintained at a density between 2 × 10^5^ and 8 × 10^5^ cells/ml by passaging every 2–3 days at 37°C and 5% CO_2_. HL-60 (ATCC) cells were cultured in RPMI 1640 medium containing 20% FBS and differentiated with complete media containing 1.5% DMSO (Sigma-Aldrich, MO, USA) for 5 days. Differentiated HL-60 (dHL-60) was used *in vitro* assay for neutrophil migration (34–36).

### Transmigration Assay

Transmigration assay was performed to confirm the reactivity of immune cells that migrated using chemotaxis. To prepare the supernatant to be placed in the lower chamber, 2 × 10^5^ of THP-1 cells were treated with MSU crystals (200 μg/ml) for 24 h, with or without PLAG, and then the cells were centrifuged to obtain only the supernatant, which was then placed in the lower chambers and the 5-μm-pore transmigration chamber (Corning, NY, USA) was placed on top. Next, a selective CXCR2 antagonist (SB225002, at 2, 10, or 40 μM; Sigma, USA), an inhibitor of CXC chemokine receptor type 1 (CXCR1, CXCR2) (reparixin, at 2, 10, or 40 μM; Medchem Express, NJ, USA), or an IL-1 receptor antagonist (IL-1RA) (2, 10, or 40 ng/ml; Peprotech, NJ, USA) were treated to differentiated HL-60 (dHL-60, 2 × 10^5^ cells), which mimics neutrophils. dHL-60 cells were placed on the upper chamber in 200 μl of serum-free RPMI 1640 medium and incubated for 24 h at 37°C. The migrated dHL-60 cells to the lower chamber were counted using a hemocytometer with trypan blue staining, and then the total number of cells in the media of the lower chamber was calculated and graphed.

### Reverse Transcription Polymerase Chain Reaction (PCR) Analysis

Total RNA was extracted from the THP-1 cells and tissue samples using Tri-RNA Reagent (Favorgen, Taiwan). The extracted RNA was used for reverse transcription (RT) with M-MLV RT reagents (Promega, Madison, WI, USA), according to the manufacturer's instructions. The primers were synthesized by Macrogen (Seoul, Republic of Korea); the primer sequences are presented in Table 1. For PCR with a specific primer pair, the synthesized cDNA was mixed with 2 × PCR Dye Mix (Bioassay) and distilled water. The amplified products were separated using 2% agarose gels, stained with ethidium bromide, and photographed under ultraviolet illumination using a WSE-5200 Printgraph 2M system (ATTO Corporation, Tokyo, Japan).

**Table 1:** RT-PCR primer sequences.

	**Forward**	**Reverse**
m.MIP-2	AGTGAACTGCGCTGTCAATG	CTTTGGTTCTTCCGTTGAGG
m.IL-1β	TGTAATGAAAGACGGCACACC	TCTTCTTTGGGTATTGCTTGG
h.CXCL8	AGGGTTGCCAGATGCAATAC	GTGGATCCTGGCTAGCAGAC
h.IL-1β	TCCAGGGACAGGATATGGAG	TCACATTCAGCACAGGACT
GAPDH	CCATCACCATCTTCCAGGAG	ACAGTCTTCTGGGTGGCAGT

### Enzyme-Linked Immunosorbent Assay (ELISA)

The concentrations of IL-1β and CXCL8 in the supernatant of the THP-1 cells were measured using the Human IL-1β and CXCL8 ELISA kit (BD Bioscience, New Jersey, USA) according to the manufacturer's instructions. The cytokine levels were estimated from a generated standard curve. The optical densities were examined at a 450-nm wavelength using a microplate reader (Molecular Devices, Sunnyvale, USA).

### Western Blot Analysis

The THP-1 cells and tissue samples were lysed on ice for 30 min using 1 × RIPA lysis buffer containing phosphatase inhibitor (Thermo Fisher Scientific Inc., MA, USA) and protease inhibitor (Roche, Basel, Switzerland). The proteins in each sample were separated using sodium dodecyl sulfate-polyacrylamide gel electrophoresis on 12% polyacrylamide gels and were blotted onto a polyvinylidene fluoride membrane (Millipore Corporation, Germany). The membrane was blocked with 5% BSA in PBS containing 0.05% Tween-20 (Calbiochem) for 1 h. It was then incubated with anti-phospho-GRK2 (Thermo Fisher Scientific Inc.), GRK2 (Santa Cruz Biotechnology), phospho-threonine (Cell Signaling Technology), α-arrestin (Cell Signaling Technology), clathrin (Abcam), β-arrestin2 (Santa Cruz Biotechnology), P2Y6 receptor (Alomone Labs), phospho-TRAM (Mybiosource, San Diego, USA), phospho-IRF3 (Ser396; Cell Signaling Technology), IRF3 (Cell Signaling Technology), IκBα (L35A5; Cell Signaling Technology), TRIF (Cell Signaling Technology), MyD88 (D80F5, Cell Signaling Technology) and β-actin (Santa Cruz Biotechnology, Dallas, Texas, USA) at 4°C overnight. After washing with PBST, the membrane was stained with goat anti-rabbit IgG peroxidase (Enzo Life Sciences, New York, USA). Target proteins were detected using Immobilon Western Chemiluminescent HRP Substrate (Millipore Corporation). For quantification of protein expression levels, we used Image J software to calculate density of the western blot bands.

### Gene Silencing by Short Interfering RNAs (siRNAs)

The specific short interfering RNAs (siRNAs) of human P2Y6 receptor (sc-42584), TRIF (sc-106845), MyD88 (sc-35986), and control siRNA (sc-37007), were purchased from Santa Cruz Biotechnology. Cells were transfected with 50 nM of target or control siRNA using HiPerFect Transfection Reagent (Qiagen, Hilden, Germany) for 24 h according to the manufacturer's protocol. The knockdown efficiencies of the siRNAs were examined using western blot analysis.

### Confocal Microscopy Examination

To detect the P2Y6 receptor on membrane surfaces, the cells were fixed using 4% paraformaldehyde (Sigma-Aldrich) and were incubated and blocked using PBS containing 1% BSA for 30 min at room temperature. Next, the cells were labeled with the rabbit anti-P2Y6 receptor antibody (1:200, APR-011; Alomone Labs, Jerusalem, Israel) for 2 h at room temperature, followed by the Alexa Flour 488 goat anti-rabbit IgG antibody (Invitrogen) for 1 h at room temperature. Lysosomal activity was determined using Lyso-Tracker (ENZ-51005, Enzo Life Sciences) according to the manufacturer's protocol. To determine the intracellular trafficking of the P2Y6 receptor, THP-1 cells were co-stained with anti-P2Y6 receptor and anti-Rab5 antibody (Santa Cruz Biotechnology) for early endosome marker. Finally, the cells were washed with 1% fetal bovine serum/PBS and mounted with 4′,6-diamidino-2-phenylindole-containing fluorescence microscopy mounting medium (Invitrogen). The samples for confocal analysis were analyzed using a laser scanning confocal microscope (Carl Zeiss, Oberkochen, Germany).

### Flow Cytometric Analysis

To detect P2Y6 receptor on membrane surfaces by FACS, the cells were fixed using 4% paraformaldehyde (Sigma-Aldrich) without permeabilization and were incubated and blocked using PBS containing 1% BSA for 30 min at room temperature. Next, the cells were labeled with the rabbit anti-P2Y6 receptor antibody (1:200; Alomone Labs) for 2 h at room temperature, followed by the Alexa Flour 488 goat anti-rabbit IgG antibody (Invitrogen) for 1 h at room temperature. Lysosomal activity was determined using Lyso-Tracker (Enzo Life Sciences) according to the manufacturer's protocol. The cells for flow cytometric analysis were washed three times with FACS buffer (PBS containing 1% FBS) and then analyzed using a FACSVerse flow cytometer (BD Biosciences). FlowJo software (Tree Star, OR, USA) was used for the data processing.

### Immunoprecipitation Assay

The samples of cells for immunoprecipitation assay were solubilized in lysis buffer containing 25 mM Tris-HCl, 150 mM NaCl, 1 mM EDTA, 1% NP-40, 5% glycerol, and protease inhibitor (Roche, Switzerland). The cell lysates were then incubated with P2Y6 receptor antibody for 16 h at 4°C, followed by incubation with 20 μl prewashed protein G-agarose beads (Roche, Switzerland) for 6 h at 4°C. The samples were washed three times and solubilized in Laemmli buffer for 10 min at 100°C and then were verified using western blot analysis.

### Statistical Analysis

The results were presented as mean ± standard deviation (SD) values. The level of significance, assumed at the 95% confidence limit or greater (*p* < 0.05), was determined using Student's *t*-tests. Statistical difference between more than two groups was analyzed by one-way ANOVA followed by Duncan's *post-hoc* test using Statistical Package for the Social Sciences 18.0 software program (IBM SPSS statistics, Hong-Kong).

### Ethics Statement

All animal experimental procedures were carried out in accordance with the Guide and Use of Laboratory Animals (Institute of Laboratory Animal Resources). All experimental procedures were approved by the Institutional Animal Care and Use Committee of the Korea Research Institute of Bioscience and Biotechnology (Daejeon, South Korea), approval number KRIBB-AEC-16155.

## Results

### PLAG Relieves Gouty Inflammation Induced With MSU

**Figure 1:**
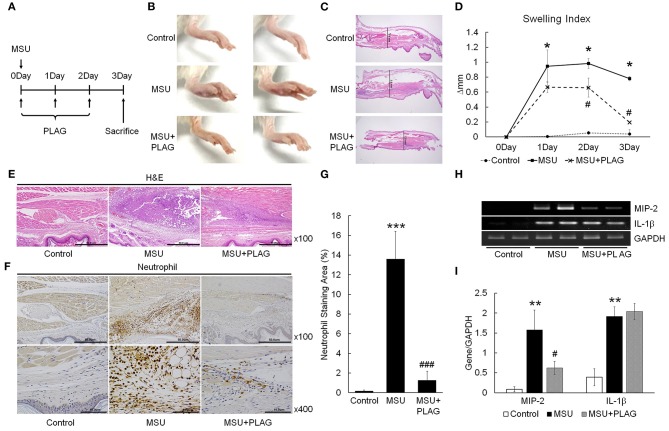
PLAG alleviates MSU-induced gouty inflammation by reducing neutrophil infiltration in gouty lesions. **(A)** BALB/c mice were divided into three groups: a PBS-treated group, an MSU-treated group, and an MSU/PLAG co-treated group. MSU (1 mg/mouse) was injected into the footpad once on 0 day and PLAG (250 mg/kg) was given via the oral route every day on 0, 1, 2, day. Photographs **(B)** and Hematoxylin and eosin (HandE)-stained sections **(C)** represents paw swelling in the MSU crystal only and MSU/PLAG-treated mice. **(D)** Footpad swelling was calculated for 3 days in the MSU crystal only and MSU/PLAG-treated mice. **(E,F)** Hematoxylin and eosin stained sections and immunohistochemical staining with anti-neutrophil antibody were performed 3 days after MSU crystal injection. **(G)** Quantification of the stained neutrophils seen in **(F)** (% of stained area). **(H)** Expression of MIP-2 and IL-1β was analyzed in the footpad tissues using RT-PCR. GAPDH was used as an internal control. **(I)** Quantification of **(H)**. Data are presented as mean ± SD. The level of significance was determined by one-way ANOVA. *p < 0.05, **p < 0.01, ***p < 0.001, compared to the control group. #*p* < 0.05, ###*p* < 0.001, compared to the MSU-treated group.

The effect of PLAG on reduction of gouty inflammation was examined using an MSU-induced acute gout animal model (Figure 1A). Swelling is an obvious sign of MSU-induced gouty inflammation and is easily observed using visual assessment. Paw swelling was observed within 1 day after injecting the MSU in the mouse footpad and was maintained for 3 days. The results of PLAG administration in the MSU-induced mice indicated that footpad swelling recovered more quickly compared with the MSU crystal-induced mice who did not receive PLAG (Figure 1B). Paw swelling was verified in the dissected tissue and the resulting thickness was measured for 3 consecutive days. The results indicated that the MSU-induced footpad swelling was maintained for 3 days in the untreated mice. But, in the PLAG-treated mice, the swelling was not severe and was resolved within 3 days (Figures 1C,D). Neutrophil infiltration in the MSU-treated regions was observed in the immunohistochemically-stained tissue. However, this neutrophil infiltration was not found in the MSU-injected footpad tissues of mice who received PLAG (Figures 1E–G). Chemokine MIP-2 expression, which is equivalent to human CXCL2, is a main factor for neutrophil recruitment into MSU-injected tissue. Chemokine MIP-2 expression was examined using RT-PCR. MIP-2 was expressed in the tissues of MSU-treated mice, but its expression was reduced in the tissues of the mice who received PLAG (Figures 1H,I). IL-1β expression was induced in the MSU-treated tissues, but PLAG had no effect on modulation of IL-1β expression in the MSU-injected tissues. Taken together, these results indicated that PLAG relieved acute gouty inflammation induced with MSU via modulation of neutrophil migration; this effect occurred within 3 days.

### PLAG Effectively Inhibits MSU-Induced Neutrophil Migration in the *in vitro* Assay

**Figure 2:**
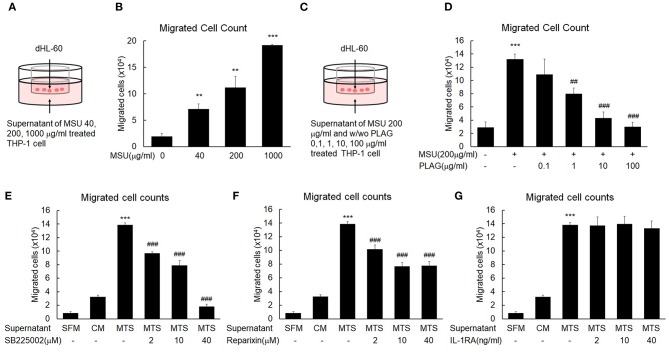
MSU-stimulated THP-1 induces neutrophil migration and PLAG attenuates the mobile activity induced by MSU. **(A)** A transwell experimental scheme for neutrophil migration by the cell-free supernatant of MSU-stimulated THP-1. The number of dHL-60 cells migrated to the bottom chamber was quantified using a hemocytometer. **(B)** Chemotaxis of dHL-60 cells in response to cell-free culture supernatants obtained from the different doses (40, 200, or 1,000 μg/ml) of MSU-treated THP-1 cells. **(C)** Transwell experimental scheme for the effect of PLAG on inhibition of neutrophil migration toward the cell-free culture supernatant of MSU-treated THP-1 cells. Different concentrations of PLAG (0.1, 1, 10, 100 μg/ml) were treated in MSU-stimulated THP-1 cells, and the cell-free supernatants were placed on the bottom chamber. dHL-60 cells were added on the upper chamber, cultured for 24 h in the culture supernatants. The transmigrated dHL-60 cells to the bottom chambers were counted using a hemocytometer. **(D)** Dose effect of PLAG on chemotaxis of dHL-60 cells in response to cell-free culture supernatants obtained from MSU-treated THP-1 cells. **(E,F,G)** dHL-60 cells were treated with SB225002 (2, 10, 40 μM) or reparixin (2, 10, 40 μM) or IL-1RA (2, 10, 40 ng/ml) and then placed on the upper chamber to see chemotaxis toward the supernatant of THP-1 cells treated with MSU (200 μg/ml). The number of transmigrated HL-60 cells was counted using a hemocytometer. Data are presented as mean ± SD. **p < 0.01, ***p < 0.001, compared to the control group. ##*p* < 0.01, ###*p* < 0.001, compared to the MSU-treated group. dHL-60, differentiated HL-60 cells; SB225002, a selective CXCR2 antagonist; reparixin, inhibitor of CXC chemokine receptor type 1 and 2; IL-1RA, interleukin 1 receptor antagonist; SFM, serum-free medium; CM, control medium; MTS, MSU-treated THP-1 supernatant.

Excessive neutrophil infiltration into tissues can cause pain and swelling (37). *In vitro* transmigration assays were performed to mimic the neutrophil transmigration in the MSU-injected tissues of the *in vivo* mouse models. Briefly, the transwell assay system consisted of an upper chamber and bottom chamber. The bottom of the upper chamber contained 5-um diameter pores that allowed factors present in the bottom chamber to move freely between the two spaces. To identify factors for neutrophil migration in the MSU-stimulated tissue cells, THP-1 cells were stimulated with 40, 200, or 1,000 μg/ml MSU. After 24 h, cells were centrifuged and cell-free supernatant was placed in the bottom chamber (Figure 2A). The upper chamber was filled with differentiated HL-60 (dHL-60) cells, which have mobile activity similar to neutrophils in the presence of chemokines. The numbers of dHL-60 cells that transmigrated from the upper to the bottom chamber were calculated after a 24-h culture period. The numbers of migrated neutrophils were increased in the culture supernatants of MSU-stimulated THP-1 cells in a dose-dependent manner (Figure 2B). To reveal the efficacy of PLAG to hinder neutrophil migration, different doses of PLAG (0.1, 1, 10, or 100 μg/ml) were applied to MSU-stimulated THP-1 cells for 24 h. The harvested cell-free supernatants of MSU-stimulated THP-1 cells, with or without PLAG, were decanted into the bottom well, the upper chamber containing differentiated HL-60 cells was put in position, and HL-60 cells that transmigrated from the upper to the bottom chamber were calculated after a 24-h incubation period (Figure 2C). The migration of dHL-60 cells with MSU stimulation was effectively reduced in the supernatants of PLAG-treated THP-1 cells in a dose-dependent manner (Figure 2D). In the supernatants of the 10 and 100 μg/ml PLAG-treated THP-1 cells, transmigration activity by MSU treatment did not occur. These results indicated that the MSU-stimulated THP-1 cells secreted factors (including chemokines) that stimulated neutrophil migration. The also indicated that PLAG effectively inhibited the increase in mobile activity that was obtained using MSU stimulation of the THP-1 cells. PLAG effectively inhibited the chemotactic activity of dHL-60 cells.

### Mobile Activity of HL-60 Cells Induced by Culture Supernatant of MSU-Stimulated THP-1 Cells Depends on Chemokine Receptors

To characterize factors that induce neutrophil recruitment into MSU-injected tissues, we stimulated THP-1 cells with MSU. Using the transwell assay we found that the supernatant of MSU-stimulated THP-1 cells contained neutrophil-attracting factors (Figure 2A). We then used SB225002 (a selective CXCR2 antagonist) and reparixin (a CXC chemokine receptor type 1 (CXCR1, CXCR2) inhibitor) to characterize the neutrophil-attracting factor. Generally, neutrophil recruitment is vital for pathogenic inflammatory reactions (38), and secreted chemokines coordinate neutrophil recruitment via CXCR1 and CXCR2 in tissues that release DAMP (39). The mobile activity of differentiated HL-60 cells was obviously increased in the cell-free supernatants of MSU-treated THP-1 cells, but this activity was gradually decreased in the treatments in which SB225002 (Figure 2E) or reparixin (Figure 2F) were added to the cell-free supernatants. These results indicated that the neutrophil transmigration activity was mainly dependent on chemokines induced in the MSU-treated macrophages. The neutrophil migration enhanced by the supernatant of MSU-treated macrophage was effectively reduced by the CXCR2 antagonists, SB225002 and reparixin (Figures 2E,F), but not by the IL-1R antagonist, IL-1RA (Figure 2G). These results indicated that the neutrophil transmigration in the MSU-treated THP-1 cells was initiated by CXCR2 ligands.

### PLAG Differentially Modulates Expression of CXCL8 and IL-1β in MSU-Treated THP-1 Cells

**Figure 3:**
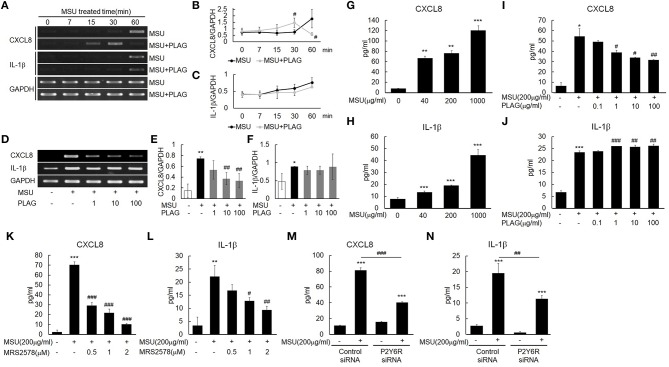
MSU binding to P2Y6 receptor induces CXCL8 and IL-1β production, and PLAG modulates CXCL8 expression but not IL-1β expression. **(A)** Time effect of PLAG on the mRNA expression of MSU-induced CXCL8 and IL-1β. THP-1 cells were pre-treated with PLAG (100 μg/ml) for 1 h and then were treated with MSU crystals (200 μg/ml). **(B,C)** Quantification of **(A)**. **(D)** Dose effect of PLAG on the mRNA expression of MSU-induced CXCL8 and IL-1β.THP-1 cells were pre-treated with different dose of PLAG (1, 10, or 100 μg/ml) and then were treated with MSU crystals (200 μg/ml). **(E,F)** Quantification of **(D)**. GAPDH was used as a control **(G,H)** Secreted CXCL8 and IL-1β were measured using ELISA in the supernatants of THP-1 cells treated with different doses of MSU crystals (50, 200, or 1,000 μg/ml). **(I,J)** Secreted CXCL8 and IL-1β in the supernatant of THP-1 cells pre-treated with different doses of PLAG (0.1, 1, 10, or 100 μg/ml) for 1h and then treated with MSU crystals (200 μg/ml) for 24 h were measured using ELISA. **(K,L)** THP-1 cells were pre-treated with MRS2578 (0.5, 1, or 2 μM) and treated with MSU crystals (200 μg/ml). Secreted CXCL8 and IL-1β in the supernatants of THP-1 cells were measured using ELISA. **(M,N)** THP-1 cells were transfected with control or P2Y6 receptor siRNA (50 nM) for 24 h. P2Y6-knockdown THP-1 cells were treated with MSU crystals (200 μg/ml) for 24 h, and secreted CXCL8 and IL-1β in the supernatants of THP-1 cells were measured using ELISA. Data are presented as mean ± SD. *p < 0.05, **p < 0.01, ***p < 0.001, compared to the control group. #*p* < 0.05, ##*p* < 0.01, ###*p* < 0.001, compared to the MSU-treated group.

IL-1β expression in MSU-treated human primary monocytes has been reported (16), and CXCL8 is induced in THP-1 cells grown with MSU (40). Our results indicated that transcripts of CXCL8 and IL-1β were expressed in the MSU-treated THP-1 cells within 60 min after MSU treatment (Figure 3A). CXCL8 and IL-1β secretion in the supernatant of MSU-treated THP-1 cells increased in a dose-dependent manner during the 24-h treatment period (Figures 3G,H). The CXCL8 mRNA and protein expression that were induced by MSU treatment were reduced in the PLAG co-treated THP-1 cells in a dose-dependent manner (Figures 3D,E,I). In the PLAG co-treated cells, CXCL8 mRNA expression was observed at 15 min and was sustained for 30 min, but it was not observed at 60 min (Figures 3A,B). The increased IL-1β mRNA and protein induced by MSU treatment was not reduced at all in the PLAG co-treated THP-1 cells (Figures 3A,C,E,F,J). These results indicated that PLAG specifically modulated CXCL8, but not IL-1β, expression.

The purinergic receptor, P2Y6, recognizes MSU in cells (16). Treatment with a selective P2Y6 antagonist, MRS2578, and an siRNA of P2Y6 verified that the expression of CXCL8 and IL-1β induced by MSU stimulation was mediated by the P2Y6 receptor. The CXCL8 and IL-1β secretion were decreased in a dose-dependent manner in the THP-1 cells treated with the antagonist (Figures 3K,L). Treatment with the siRNA against P2Y6 also reduced the secretion of CXCL8 and IL-1β (Figures 3M,N). These results suggested that the expression of CXCL8 and IL-1β was mainly mediated by the purinergic receptor, P2Y6.

### MSU-Induced Endocytosis and Exocytosis Cycle of P2Y6 Receptor Is Accelerated in PLAG-Treated Cells

**Figure 4:**
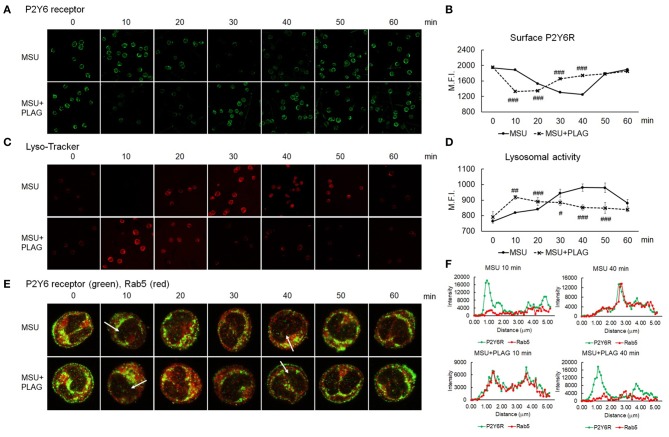
Accelerated intracellular trafficking of MSU-loaded P2Y6 in PLAG-treated cells. **(A)** Analysis of cell surface P2Y6 receptor using confocal microscopy. THP-1 cells were pre-treated with PLAG (100 μg/ml) for 1 h and then were treated with MSU (200 μg/ml). The cells were harvested and were fixed with 4% paraformaldehyde. The membrane-localized receptor was stained with anti-P2Y6 receptor antibody without permeabilization. **(B)** The result of **(A)** was also analyzed by flow cytometry. **(C)** Lysosomal activity was measured using lyso-ID and analyzed using confocal microscopy. **(D)** The result of **(C)** was also analyzed by flow cytometry. **(E)** Co-localization of P2Y6 receptor and Rab5 was examined using confocal microscopy. **(F)** Graphs show fluorescence intensity profiles calculated on the white arrows of the confocal images in **(E)** using ZEN program. Data are presented as mean ± SD. #*p* < 0.05, ##*p* < 0.01, ###*p* < 0.001, compared to the MSU-treated group.

Since we found out that MSU-induced CXCL8 and IL-1β are produced through P2Y6 receptor, we next investigated how PLAG regulates the activation of P2Y6 receptor. Intracellular trafficking of DAMP-loaded GPCR is known to be associated with cellular signaling and recycling of GPCR (22, 41). Therefore, we examined trafficking of MSU-stimulated P2Y6 receptor with or without PLAG treatment. Intracellular trafficking of P2Y6 receptor began at 20 min, and returned to the membrane at 50 min, after MSU treatment (Figures 4A,B). In PLAG co-treated cells, endocytosis of P2Y6 receptor occurred at 10 min, and the receptor reappeared to the membrane at 30 min (Figures 4A,B). We next checked the cycle of endocytosis using an endosome staining dye, Lyso-Tracker. We observed that MSU crystals induced the initiation of endocytosis at 20 min and the termination at 50 min. PLAG significantly accelerated the endosomal activity as observed at 10 min after MSU crystal treatment (Figures 4C,D). Endosomal trafficking of P2Y6 was further verified through a co-localization assay using Rab5, an early endosome marker. During intracellular trafficking of P2Y6 receptor, co-localization of Rab5 was observed as a yellow color at 30–40 min after MSU treatment. In PLAG pretreated cells, co-localization was observed at 10 min and dissociation occurred at 30 min (Figures 4E,F). These results indicates that PLAG effectively accelerated the intracellular trafficking of MSU-loaded P2Y6 receptor.

### PLAG Induces GRK2 Phosphorylation, Which Enables Acceleration of Intracellular Trafficking of the P2Y6 Receptor

**Figure 5:**
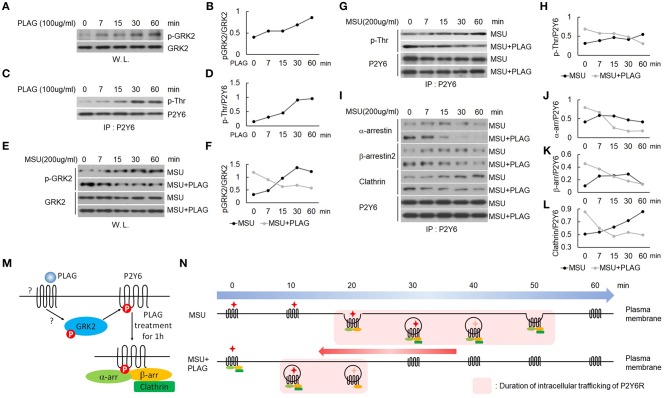
PLAG phosphorylates GRK2 and P2Y6 receptor to form a receptor endocytosis-related protein complex. **(A)** Effect of PLAG on phosphorylation of GRK2. THP-1 cells were treated with 100 μg/ml PLAG for 0, 7, 15, 30, and 60 min. Whole lysates were used to analyze phosphorylated GRK2 and total protein of GRK2. **(B)** Quantification of **(A)**. **(C)** Effect of PLAG on phosphorylation of P2Y6 receptor. Cell lysates were immunoblotted with phospho-threonine antibody after immunoprecipitation with the P2Y6 receptor antibody. **(D)** Quantification of **(C)**. **(E)** THP-1 cells were incubated with 100 μg/ml PLAG for 1 h and then were stimulated with 200 μg/ml MSU crystals for 0, 7, 15, 30, and 60 min. Whole lysates were used to analyze phosphorylated GRK2 and total protein of GRK2. **(F)** Quantification of **(E)**. **(G)** Cell lysates were immunoblotted with phospho-threonine antibody after immunoprecipitation with the P2Y6 receptor antibody. **(H)** Quantification of **(G)**. **(I)** Cell lysates were immunoblotted with α-arrestin, β-arrestin2, or clathrin antibodies after immunoprecipitation with the P2Y6 receptor antibody. **(J,K,L)** Quantification of **(I)**. **(M)** Schematic diagram of endocytosis-related protein complex formation by PLAG treatment for 1 h. One hour after PLAG treatment, GRK2 and P2Y6R were phosphorylated, and endocytosis-related proteins such as α-arrestin, β-arrestin, and clathrin were complexed with P2Y6R. **(N)** Schematic diagram of P2Y6 receptor endocytosis and duration of intracellular trafficking in MSU-treated THP-1 cells. PLAG, using a preformed endocytosis-related complex, promotes the initiation of intracellular trafficking of P2Y6R and rapidly brings it back to the cell surface when MSU is treated. Therefore, PLAG shortens the duration of P2Y6R stays in the cytoplasm.

We verified that PLAG accelerated intracellular trafficking of MSU-stimulated P2Y6 receptor in Figure 4. Agonist-dependent GPCR phosphorylation is known to be mediated by GRK, which induces binding of arrestins to the receptor for endocytosis (42). Endocytosis of the P2Y6 receptor is initiated by GRK2-induced receptor phosphorylation (19, 43). Phosphorylation of GRK2 was increased in PLAG-treated THP-1 cells (Figures 5A,B) and, subsequently, the threonine residues of P2Y6 phosphorylation were detected (Figures 5C,D). When pretreated with PLAG 1 h, the already phosphorylated GRK2 and P2Y6 receptor was rapidly dephosphorylated by MSU treatment, while MSU crystals alone gradually induce the phosphorylation of the proteins (Figures 5E–H). In the same manner, PLAG pretreatment already induced the binding of α-arrestin, β-arrestin 2, and clathrin to P2Y6 receptor as observed at 0 min, and rapidly detached the proteins from the receptor when MSU crystals treated (Figures 5I–L). Taken together, these results indicate that PLAG accelerated internalization of MSU-loaded P2Y6 receptor via GRK2 phosphorylation and promoted recruitment of α-arrestin, β-arrestin 2, and clathrin (Figure 5M). PLAG accelerated intracellular trafficking of MSU-stimulated P2Y6 receptor through enhanced endocytosis and exocytosis (Figure 5N).

### The Signal Pathway for CXCL8 Expression Mainly Relies on Endocytosed P2Y6 Receptor and PLAG Effectively Attenuates the Endosome-Dependent Signal Pathway

**Figure 6:**
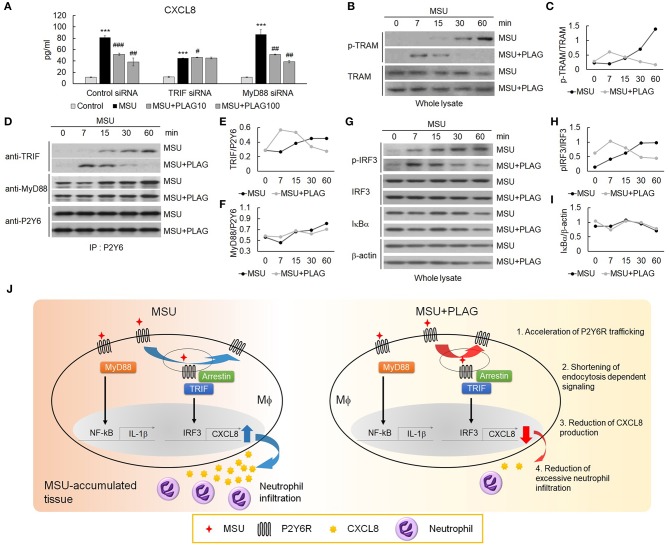
The reduction of CXCL8 expression by PLAG is dependent on TRIF/IRF3 signaling. **(A)** THP-1 cells were transfected with TRIF and MyD88 siRNA. After 24 h, the cells were incubated with 100 μg/ml PLAG or DMSO for 1 h. The cells were then treated with 200 μg/ml of MSU crystals for 24 h. The culture supernatants were harvested and assayed using ELISA to check the secreted levels of CXCL8. **(B)** THP-1 cells were incubated with 100 μg/ml PLAG or DMSO for 1 h and were stimulated with 200 μg/ml MSU crystals for 7, 15, 30, and 60 min. Whole lysates were used to analyze the phosphorylation of TRAM. TRAM was used as the loading control. **(C)** Quantification of **(B)**. **(D)** TRIF signaling during trafficking was evaluated in THP-1 cells. Cells were incubated with 100 μg/ml PLAG or DMSO for 1 h and were stimulated with 200 μg/ml of MSU crystals for 7, 15, 30, and 60 min. Cell lysates were immunoblotted with TRIF and MyD88 antibodies after immunoprecipitation with the P2Y6 receptor antibody. **(E,F)** Quantification of **(D)**. **(G)** Whole lysates were used to analyze the phosphorylation of IRF-3 or IκBα degradation. β-Actin was used as a loading control. **(H,I)** Quantification of **(G)**. **(J)** A schematic diagram showing the mechanism of action of PLAG. PLAG promotes MSU-induced P2Y6 receptor intracellular trafficking (1) and reduces the duration of endocytosis-dependent signaling (2). Consequently, PLAG reduces MSU-induced CXCL8 production (3) and prevents excessive neutrophil recruitment into MSU-accumulated tissue (4). Data are presented as mean ± SD. ***p < 0.001, compared to the control group. #*p* < 0.05, ##*p* < 0.01, ###*p* < 0.001, compared to the MSU-treated group.

Endocytosis of GPCR is regarded as the termination of G-protein-mediated cellular signaling (44). However, internalized GPCRs are still in an active state for transmitting signals from the receptor-anchored endosomes (45). The endosome-dependent signals have different cellular functions from the signals transmitted by the receptor in the membrane (46). Therefore, we further investigated signaling molecules associated with CXCL8 expression through the use of small interfering RNAs (siRNA) of TIR-domain-containing adapter-inducing interferon-β (TRIF) or myeloid differentiation primary response 88 (MyD88) introduced to THP-1 cells. In siRNA transfected TRIF-silenced cells, CXCL8 expression was reduced by up to 50% compared with control siRNA transfection, but not in MyD88-silenced cells (Figure 6A). Moreover, PLAG reduced CXCL8 expression in a dose-dependent manner in the scrambled RNA and MyD88-silenced cells, but not in the TRIF-silenced cells (Figure 6A). These results indicate that MSU-induced CXCL8 expression mainly relies on TRIF signals, and PLAG regulates the modulation of only TRAM/TRIF-dependent signaling. Endosomal localization of TRAM is essential for Toll-like receptor-mediated type I IFN-inducing signals from the endosome (47, 48). Next, we investigated whether PLAG regulates TRAM signaling in whole lysates of THP-1 cells. As a result, PLAG accelerated MSU-induced phosphorylation of TRAM (Figures 6B,C). The result of the immunoprecipitation assay using anti-P2Y6 indicated that advanced association of TRIF with P2Y6 was also detected in MSU and PLAG co-treated cells, but no effect in the association of MyD88 with P2Y6 (Figures 6D–F). Subsequently, the advanced activity of IRF3 (examined by western blot analysis using anti-phospho-IRF3) was detected in MSU and PLAG co-treated cells, while no effect on IκBα degradation (Figures 6G–I). Taken together, these results indicate that PLAG accelerated MSU-induced internalization and returning to the membrane of P2Y6 receptor. During trafficking of P2Y6 receptor, the activation of signaling molecules leading to CXCL8 expression (i.e., TRAM/TRIF and transcriptional factor activity of IRF3) was advanced and shortened. This process resulted in the reduction of CXCL8 expression (Figure 6J).

### Specificity of PLAG in the Promoted Endocytosis of P2Y6 Modulation of CXCL8 Expression

**Figure 7:**
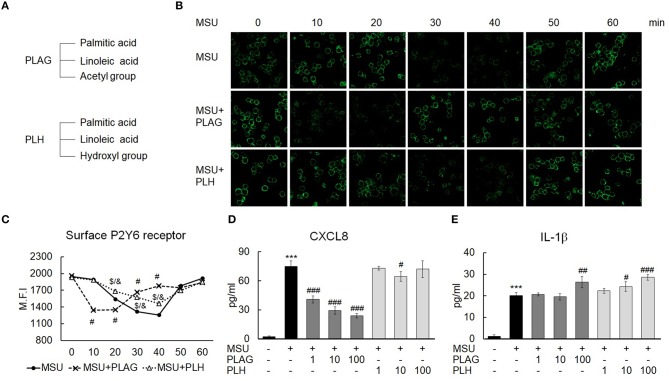
Specificity of PLAG in accelerated P2Y6 endocytosis and the modulation of CXCL8 expression. **(A)** Chemical structure of PLAG and PLH. **(B)** Advanced endocytosis by PLAG was compared with that by PLH. Cells were incubated with 100 μg/ml PLAG, PLH, or DMSO for 1 h and then stimulated with 200 μg/ml of MSU crystals. The cells were harvested over time were fixed using 4% paraformaldehyde. P2Y6 receptor expressed on the membrane was stained with the P2Y6 receptor antibody without permeabilization and was analyzed using confocal microscopy. **(C)** The result of B was also confirmed by flow cytometry. **(D,E)** PLAG was compared with PLH to check the secretion levels of CXCL8 and IL-1β. The cells were incubated with 100 μg/ml PLAG, PLH, or DMSO for 1 h and were treated with 200 μg/ml MSU crystals for 24 h. The culture supernatants were harvested and used for ELISA. Data are presented as mean ± SD. ***p < 0.001, compared to the control group; #*p* < 0.05, ##*p* < 0.01, ###*p* < 0.001, statistical difference between the MSU-treated group and MSU + PLAG treated group; $p < 0.05, statistical difference between the MSU-treated group and MSU + PLH treated group; &*p* < 0.05, statistical difference between the MSU + PLAG-treated group and MSU + PLH-treated group.

PLAG and PLH are diacylglycerols containing two acyl groups, palmitic and linoleic acid, at the 1, 2-positions on the glycerol backbone. PLAG and PLH have an acetyl group and a hydroxyl group, respectively, at the 3-position (Figure 7A). In the MSU-treated THP-1 cells, P2Y6 intracellular trafficking was initiated at 30 min, and the receptor returned to the membrane at 50 min. This approximately 30-min trafficking duration indicated that the P2Y6 receptor was endosomal for 30 min. In the PLAG-treated cells, P2Y6 internalization was advanced and an earlier return of P2Y6 to the membrane occurred. However, in the MSU/PLH-treated cells, P2Y6 intracellular trafficking showed the same phenotype as that of the cells treated with only MSU. This result indicated that PLAG had specific biological activity during advanced endocytosis and return of the P2Y6 receptor to the membrane in the MSU-treated cells (Figures 7B,C). CXCL8 expression induced by MSU was attenuated by PLAG but not by PLH (Figure 7D). PLAG and PLH had no effect on modulation of IL-1β expression (Figure 7E). These results indicated that the shortened endosome duration of the P2Y6 receptor and attenuation of CXCL8 expression were specific to the biological efficacy of PLAG and did not occur during PLH treatment.

## Discussion

In the previous study, PLAG induced rapid resolution of inflammation in LPS-induced acute lung injury via early elimination of LPS and early termination of endocytosis-dependent signaling of TLR4 (49). PLAG remarkably ameliorated LPS-induced acute lung injury, which is a disease caused by a PAMP molecule (49). In this study, we examined a mitigating effect of PLAG on gouty inflammation, one of the sterile inflammations caused by MSU crystal, a substance thought to be DAMP. Neutrophil infiltration to the tophi is a major cause of the pain in the gouty tissues (50, 51), and neutrophils are recruited by chemotactic activity in response to neutrophil-attracting chemokines, such as CXCL8, which are produced in MSU-accumulated tissues. Therefore, modulating the chemokine production may be a new therapeutic target to reduce the pain of acute gout. We observed that PLAG effectively reduced MSU-induced footpad swelling and neutrophil infiltration by modulating MIP-2 (CXCL8) production in a mouse model of MSU-induced acute gout.

In the development of gouty inflammation, various types of cells and molecular pathways are involved. Of which, neutrophil-macrophage interaction plays an important role in the acute inflammatory response (8). Gouty inflammation begins at where monocytes and macrophages first contact with MSU crystals. When these immune cells are stimulated by MSU, they secrete pro-inflammatory cytokines and chemokines to attract neutrophils to the gouty region. Especially, IL-1β and CXCL8 are the most typical factors associated with the progression of the disease. IL-1β is a critical factor in that gout is primarily initiated by this cytokine. Insoluble crystals of MSU accumulated in the joints activate the NLRP3 inflammasome to secrete IL-1β. This cytokine binds to IL-1 receptor type I (IL-1RI) to recruit MyD88 through the TIR domain, which consequently induces gene expression and secretion of pro-inflammatory cytokines. In addition, CXCL8, also known as a neutrophil chemotactic factor, is a typical chemokine produced in the progression of gout, which responsible not only for inducing neutrophils to migrate toward the gout lesion, but also for stimulating the cells to release neutrophil extracellular traps (NETs) through the CXCL8/CXCR2 axis. In this study, we demonstrated that PLAG ameliorated MSU-induced gouty inflammation by specifically regulating transcriptional and translational expression of CXCL8, without affecting IL-1β gene expression and secretion.

The purinergic P2Y6 receptor is a G protein-coupled receptor (GPCR) which has a higher affinity for the nucleotide uridine diphosphate (UDP) than other nucleotides. It has been demonstrated that UDP stimulates P2Y6 receptor to release CXCL8 in human THP-1 monocytic cells (52). In recent years, however, many studies have reported that MSU stimulates P2Y6 receptor expressed in normal human keratinocytes and monocytes to produce pro-inflammatory cytokines including CXCL8, IL-1α and IL-6 in MSU-associated inflammatory diseases (16). In addition, the P2Y6 receptor antagonist MRS2578 inhibited neutrophil activation and NET release induced by MSU crystals. In the same way, we confirmed that MRS2578 inhibits both CXCL8 and IL-1β expression, and PLAG selectively reduces P2Y6 receptor-mediated CXCL8 production induced by MSU crystals.

The selectivity of PLAG in the regulation of MSU-induced CXCL8 expression is associated with its control ability of P2Y6 receptor endocytosis. Previously, we demonstrated that PLAG accelerates the termination of LPS-induced TRAM and TRIF-dependent TLR4 signaling, resulting in the reduced IRF3-dependent CXCL8 expression (49). In the study, PLAG did not affect MyD88-mediated NF-κB activation that induces the transcriptional expression of IL-1β. In the same manner, we observed that PLAG accelerates the termination of P2Y6 receptor-mediated endocytosis by promoting earlier binding of β-arrestin-1 and −2 and clathrin to the receptor, which subsequently leads to earlier dissociation of the molecules from the receptor. We also verified that the internalized P2Y6 receptor to the cytoplasm recruits the adapter molecules TRAM and TRIF to activate IRF3-dependent CXCL8 expression, and PLAG effectively advanced the phosphorylation of these molecules followed by earlier termination of the signaling pathway. On the other hand, PLAG did not affect MSU-induced IL-1β gene expression and protein secretion, which is produced through MyD88/NF-κB signaling and NLRP3 inflammasome activation (53, 54). From these observation, besides TLR4, we discovered that PLAG has a regulatory effect on the endocytosis of a member of GPCR family, P2Y6, and endocytosis-dependent signaling pathways.

The current therapeutic agents used for gout treatment are nearly focused on reducing IL-1β production or blocking IL-1β-mediated signaling pathways and gene expression (55). Anakinra is a recombinant non-glycosylated IL-1 receptor antagonist (IL-1RA) used for gouty inflammation and rheumatoid arthritis. It works by inhibiting the interaction between IL-1 receptor and its ligands IL-1α and IL-1β (56–58). Colchicine, under the brand name of Colcrys, is another gout drug approved by FDA as the first single-ingredient oral product. Basically, this drug works by blocking the formation of microtubules by binding to α- and β-tubulin (59, 60). In gouty inflammation, colchicine inhibits microtubule-mediated inflammatory pathway that induces the activation of NLRP3 inflammasome and IL-1β secretion (61). However, repeated colchicine treatment induces bone marrow suppression, and intracellular accumulation of colchicine leads to toxic effect, such as abdominal pain, unusual bleeding, muscle weakness, or pain (62, 63). Thus, there are limitation on the usage and dose of administration of this drug. Unlike the two drugs mentioned above, PLAG showed the therapeutic effects by down-regulating MSU-induced CXCL8 production. It was demonstrated that CXCL8 levels are more highly increased than other pro-inflammatory cytokines in gout patients, and it is directly associated with neutrophil infiltration, pain, and the development of chronic gouty arthritis (64, 65). Therefore, we think that CXCL8 is also a good therapeutic target for the treatment of gouty inflammation. In addition, there were no reports of toxicity or adverse effects when high doses of PLAG were administered in the non-clinical toxicity test (49, 66). Since PLAG is delivered via oral administration, it has an advantage in the way of drug delivery than other gout drugs.

Macrophages and monocytes play important roles in both driving and resolving gouty inflammation. These immune cells secrete pro-inflammatory cytokines and chemokines, such as IL-1α, IL-1β, IL-6, TNF, and CXCL8 to induce acute phase of inflammation. For resolution of gouty inflammation, macrophages perform essential process called efferocytosis to eliminate dead neutrophils and aggregated NETs. We already verified that PLAG promotes macrophage efferocytosis by enhancing macrophage mobility via membrane redistribution of P2Y2 (67). From the results, we expect that PLAG can help resolving of acute gouty inflammation by promoting efferocytosis. Direct evidences on the role of PLAG in efferocytosis during MSU crystal-induced gouty inflammation should be investigated.

NETs, produced by NETosis of neutrophils, are a major pathogenesis of gout. Netosis is very effective process to eliminate DAMP molecules including MSU for resolution of inflammation. But prolonged NETosis can evoke tissue injury, which can cause severe and chronic gouty inflammation. In this paper, we focused on the role of PLAG on the modulation of neutrophil recruitment in MSU-introduced inflammatory lesion. We think that the reduced recruitment of neutrophil into the lesion by PLAG could be helpful to reduce excessive NET formation.

In summary, as shown in Figure 6J, PLAG showed its therapeutic efficacy by reducing chemotaxis-dependent recruitment of neutrophils into MSU-induced gouty lesions. In cellular levels, PLAG promoted MSU-induced P2Y6 receptor intracellular trafficking by reducing the duration of endocytosis-dependent signaling, resulting in the reduced production of CXCL8. Therefore, we think that PLAG could be a promising therapeutic agent for the treatment of gouty inflammation.

## Data Availability Statement

The raw data supporting the conclusions of this article will be made available by the authors, without undue reservation, to any qualified researcher.

## Ethics Statement

The animal study was reviewed and approved by Institutional Animal Care and Use Committee of the Korea Research Institute of Bioscience and Biotechnology.

## Author Contributions

JWK and S-HS: conception and design. S-HS and JHK: development of methodology and acquisition of data. JWK, S-HS, JHK, and SY: analysis and interpretation of data. JWK, JJ, SY, and S-HS: writing and review of the manuscript. K-YS: supporting and provision of materials.

## Conflict of Interest

JJ, K-YS, and SY were employed by ENZYCHEM Lifesciences (Seoul, Republic of Korea). The remaining authors declare that the research was conducted in the absence of any commercial or financial relationships that could be construed as a potential conflict of interest.
